# Effects of Kombucha on oxidative stress induced nephrotoxicity in rats

**DOI:** 10.1186/1749-8546-4-23

**Published:** 2009-11-27

**Authors:** Ola Ali Gharib

**Affiliations:** 1 Drug Radiation Research Department, National Centre for Radiation Research and Technology, Atomic Energy Authority, Nasr City, Cairo, Egypt

## Abstract

**Background:**

Trichloroethylene (TCE) may induce oxidative stress which generates free radicals and alters antioxidants or oxygen-free radical scavenging enzymes.

**Methods:**

Twenty male albino rats were divided into four groups: (1) the control group treated with vehicle, (2) Kombucha (KT)-treated group, (3) TCE-treated group and (4) KT/TCE-treated group. Kidney lipid peroxidation, glutathione content, nitric oxide (NO) and total blood free radical concentrations were evaluated. Serum urea, creatinine level, gamma-glutamyl transferase (GGT) and lactate dehydrogenase (LDH) activities were also measured.

**Results:**

TCE administration increased the malondiahyde (MDA) and NO contents in kidney, urea and creatinine concentrations in serum, total free radical level in blood and GGT and LDH activities in serum, whereas it decreased the glutathione (GSH) level in kidney homogenate. KT administration significantly improved lipid peroxidation and oxidative stress induced by TCE.

**Conclusion:**

The present study indicates that Kombucha may repair damage caused by environmental pollutants such as TCE and may be beneficial to patient suffering from renal impairment.

## Background

Kombucha is a sour beverage prepared from the fermentation of black tea and sugar with a symbiotic culture of acetic acid bacteria and yeasts such as *Bacterium xylinum, Bacterium xylinoides, Bacterium gluconicum, Saccharomyces ludwigii, Saccharomyces apiculatus varieties, Schizosaccaromyces pombe, Acetobacter ketogenum*, *Torula varieties*, *Pichia fermantans *and other yeasts reported to have potential health effects [1]. Fermentation and oxidation processes of Kombucha microorganisms produce a wide range of organic acids, vitamins and enzymes. Research indicated that Kombucha improved resistance against cancer, prevented cardiovascular diseases, promoted digestion, stimulated immunity and reduced inflammation [2].

Glucuronic acid is one of the organic acids produced during fermentation process in Kombucha and may improve oxidative metabolism [3]. Trichloroethylene (TCE) is a major environmental contaminant and an occupational concern due to its widespread industrial use [4]. An animal carcinogen, TCE is nephrotoxic and causes renal tumors in rats [5]. The toxicity of TCE is dependent on its reactive metabolites derived from the reaction of glutathione conjugating with TCE, followed by subsequent metabolism by gamma-glutamyl transferase (GGT), dipeptidases and cystein conjugate B-layse [6]. Previous studies found significant renal dysfunction in male Sprague Dawley rats exposed to TCE. The renal dysfunction was manifested by glycosuria and alterations in plasma creatine, urine nitrogen, uric acid and creatine clearance, concentration related changes in hematocrit and erythrocytes, as well as reticulocyte and erythroblast counts [7,8].

TCE induced oxidative stress [9] which is considered an imbalance between the production of oxidizing molecular species (free radicals) and the presence of cellular antioxidants [10]. Containing unpaired electron, free radicals are highly reactive and cause damage to part of cells by inducing DNA strand breaks, purine oxidation and protein DNA cross linking and cell membrane damage [11]. Accumulation of such damage may cause cell death [12].

Wang *et al*. [13] reported that TCE exposure not only increased lipid peroxidation but also accelerated autoimmune responses. Lash *et al*. documented that kidney cells from male rats are more sensitive to TCE than those from female rats or hepatocytes from rats of either sex [14]. Moreover acute renal cellular injury from TCE is believed to be associated with metabolites derived from the GSH conjugation pathway [6]. The first step involve conjugation with GSH that catalyze by the GSH transferase to form the GSH conjugate DCVG and processing of the GSH conjugate by GGT and dipeptidase activities to generate the cystein conjugate S-(1,2-dichlorovinyl) L. cystein (DCVC) [14]. DCVC may also undergo sulfoxidation to form S-(1-chloro-2-(S-glutathionyl)-L-cystein sulfoxide (DCVC sulfoxide), which is a potent nephrotoxicant in rat kidney cells [15].

The present study aims to investigate the antioxidant properties of Kombucha constituents and the protective effects of Kombucha on the kidney of TCE-treated rats.

## Methods

### Animals

Twenty male albino rats weighing 150-200 g were purchased from the Egyptian Organization for Biological Products and Vaccines (Cairo, Egypt). Animals were housed in cages with good ventilation and illumination and provided with standard diet and water *ad-libitum*. All procedures in the present study conform to international animal care guidelines and the ethics committee of the institution.

### Chemicals

Analytical-grade TCE was purchased from El-Nasr Pharmaceutical Chemical (Egypt). All other chemicals and bio-chemicals were obtained from Sigma Chemical (USA). The kits used in the experiments were purchased from Bio-Diagnostics (UK).

### Preparation of Kombucha

One hundred grams (100 g) of sugar was added to one liter (1 L) of distilled water, and the solution was boiled for 15 minutes in a sterile conical flask. Six tea bags of black tea powder (Lipton, Egypt) were added to the flask (12 g/L, 1.2%) and allowed to cool to room temperature for one hour.

### Fermentation

Kombucha culture was kept under aseptic conditions. Fermentation was carried out by incubating the Kombucha culture at 28 ± 1°C for 8-10 days. Subsequently, the medium (brew) was centrifuged at 3000 rpm for 30 minutes aseptically and stored in polypropylene vials at -20°C for further use [16].

### Study design

Rats were divided into four groups (5 rats per group), namely the control group, Kombucha (KT) group, TCE group and KT/TCE group. In the control group, animals (*n *= 5) were gavage fed with maize oil (vehicle of TCE) for ten consecutive days. In the KT group, animals (*n *= 5) were administered with KT ferment per oral (0.1 ml per 100 g of body weight) for two weeks [17]. In the TCE group, animals (*n *= 5) were administrated with TCE (1 g per kg of body weight) per oral for ten consecutive days [18]. In the KT/TCE group, animals (*n *= 5) were administered with KT ferment first for two weeks and subsequently gavage fed with TCE for ten consecutive days. Animals were sacrificed 24 hours after TCE administration. Kidneys were removed. Serum was isolated for the assessment of kidney functions.

### Total free radicals assay by electron spin resonancetechnique (ESR)

Electron spin resonance occurs when a spinning electron in an externally applied magnetic field absorbs sufficient electromagnetic radiation to cause inversion of electrons spin state (e.g. transfer from ground state to excited state). This technique is used to study free radical concentrations in biological materials by detecting the molecules with unpaired electrons (free radicals) without destroying them. Free radicals from biological materials such as reactive oxygen species (O2^-^), hydroxyl radical (OH^-^), nitrogen oxide (NO^-^) and hypochlorous acid (HOCL^-^) are responsible for certain diseases [19].

### Preparation of lyophilized blood samples for ESR

Blood samples were lypophilized in a super Modulyo freeze dryer (Edwards Vacuum, UK).

### ESR spectrometer

ESR or electron paramagnetic resonance (EPR) signals were recorded at room temperature by a Bruker EMX spectrometer (X band, Bruker, Germany). ESR detection limits (1013 spins/g) depend on the sample type, sample size, detector sensitivity, frequency of incident radiation and electronic circuit of the instrument.

### Measurement and analysis of ESR spectra

Samples were inserted into the EPR of quartz tubes and measured at suitable instrument parameters. The peak height of the radiation-induced EPR signals was determined for each sample. The reading intensities were divided by the weight of each sample for normalization. To monitor variation in the peak height EPR signals as a function of magnetic field, we measured intensities as the distance between top and bottom points of the first derivative and the reading intensities were divided by sample weight of each sample for the calculation of normalization values which were recorded according to Gohn [20] and Pascual *et al*. [21].

### Biochemical assays

All biochemical assays were performed with a Helios Thermo-Spectronic spectrophotometer (Thermo Spectronic, UK). Lactate dehydrogenase (LDH) activity was evaluated according to the method by IFCC [22]. GGT activity was evaluated according to the method by Szasz [23]. Urea concentration was measured according to the method by Halled and Cook [24] with a Bio-Diagnostic kit. Creatinine level was measured according to the method by Henery [25] with a Bio-Diagnostic kit. Total protein of serum and kidney was measured according to the method by Gomal *et al*. [26]. Concentration of kidney malondialdehyde (MDA) was analyzed according to the method by Yoshioka *et al*. [27]. Kidney homogenate of both GSH content was measured according to the method by Beutler *et al*. [28]. Nitric oxide (NO) concentration was measured according to the method by Geng *et al*. [29].

### Statistical analysis

Quantitative data were expressed as mean ± SD (standard deviation) and analyzed by one way analysis of variances (ANOVA) followed by Tukey's multiple comparison test. Statistical analysis was performed with the GraphPad software (USA). Differences were considered statistically significant when *P *< 0.05.

## Results

In the present study, kidney protection effects of KT were investigated through kidney functions affected by carcinogen, e.g. serum urea, creatinine concentration, LDH and GGT activity.

### TCE administration

TCE administration significantly increased urea (*P *< 0.001) and creatinine (*P *< 0.01) levels in rats (Table 1). Administration of TCE induced a marked oxidative stress measured by lipid peroxidation (*P *< 0.001) and significant inhibition in GSH content (*P *< 0.01).

**Table 1:** Effects of KT and TCE administration on serum urea (mmol/l) and creatinine (mg/dl) concentrations 24 hours after last treatment (*n *= 5)

Treatment	Urea concentration (mmol/l)	Creatinine concentration (mg/dl)
Control	3.635 (0.388)	0.864 (0.0921)
KT	3.896 (0.34)	0.824 (0.1315)
TCE	7.381 (0.881) ^(a, b)^	1.112 (0.0867) ^(a, b)^
KT+TCE	5.794 (1.492) ^(a, b, c)^	0.952 (0.0335)

Data are presented as mean (SD). ^(a) ^Significantly different from the control group (*P *< 0.05). ^(b) ^Significantly different from the KT group (*P *< 0.05). ^(c) ^Significantly different from the TCE group (*P *< 0.05). KT: Kombucha, TCE: trichloroethylene.

**Figure 1:**
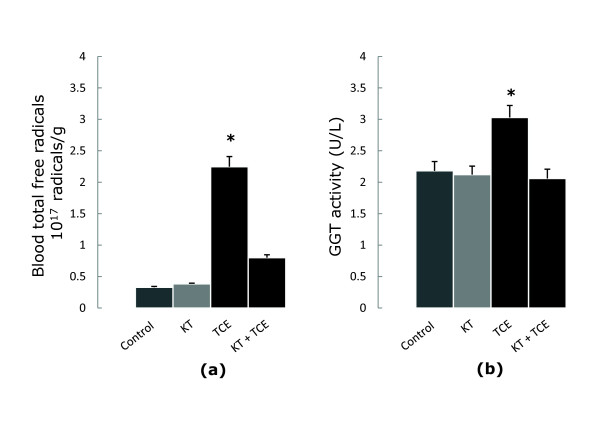
**Effects of KT and TCE administration on blood total free radicals 24 hours after last treatment (*n *= 5)**. *Significantly different from the control group (*P *< 0.05). KT: Kombucha. TCE: trichloroethylene.

Serum LDH activity (*P *< 0.001) and kidney NO concentration (*P *< 0.001) were significantly increased (Table 2). TCE administration significantly increased total free radicals in blood (*P *< 0.001) and in serum GGT activity (*P *< 0.01) (Figure 1).

**Table 2:** Effects of KT and TCE administration on serum LDH (U/L) activity and kidney NO (μmol/g protein) concentration 24 hours after last treatment (*n *= 5)

	LDH activity (U/L)	NO concentration (μmol/g protein)
Control	377.6 (39.72)	19.78 (1.808)
KT	366.2 (44.65)	21.93 (2.095)
TCE	551.2 (68.89) ^(a, b)^	39.08 (6.562) ^(a, b)^
KT+TCE	395.6 (32.19) ^(c)^	29.22 (4.181) ^(a)^

Data are presented as mean (SD). ^(a) ^Significantly different from the control group (*P *< 0.05). ^(b) ^Significantly different from the KT group (*P *< 0.05). ^(c) ^Significantly different from the TCE group (*P *< 0.05). KT: Kombucha, TCE: trichloroethylene.

### Recovery

Data of kidney GSH (Table 3) and LDH and NO concentration (Table 2) showed that KT administration restored these parameters to normal values in TCE-treated rats. Moreover, a significant improvement in serum creatinine and kidney MDA was observed (Tables 1 and 3).

**Table 3:** Effects of KT and TCE administration on kidney MDA (μmol/g protein) concentration and GSH (mg/g protein) content 24 hours after last treatment (*n *= 5)

	MDA concentration (μmol/g protein)	GSH content (mg/g protein)
Control	22.020 (4.385)	1.242 (0.0471)
KT	21.800 (2.142)	1.416 (0.1711)
TCE	36.13 (1.461) ^(a, b)^	0.908 (0.0814) ^(a, b)^
KT+TCE	27.300 (3.179) ^(b, c)^	1.229 (0.1794) ^(c)^

Data are presented as mean (SD). ^(a) ^Significantly different from the control group (*P *< 0.05). ^(b) ^Significantly different from the KT group (*P *< 0.05). ^(c) ^Significantly different from the TCE group (*P *< 0.05). KT: Kombucha, TCE: trichloroethylene.

## Discussion

The present study confirms the findings of Goel *et al*. and Khan *et al*. that TCE significantly increased urea and creatinine in rats [30,31] and that TCE also increased the activity of LDH as reported by Lash *et al*. [32]. Moreover, oxidative markers measured as lipid peroxidation in kidney tissue and total free radicals in blood increased markedly followed by a decrease in kidney glutathione content. The present study confirms the previous study [6] that GGT was increased due to TCE administration. Furthermore, the present study shows that the depletion of GSH enhances utilization of protein thereby increasing the urea level that is accompanied by an increased creatinine level suggested by Mostafa [33].

Kombucha is a potent antioxidant demonstrated to reduce the damage induced by oxidative stress [16,27,34-36]. Results from the present study show that Kombucha ferment ameliorated TCE-induced kidney damage, attributable to acetic acid which is capable of conjugating with toxins, solubilizing and eliminating them from the body [37]. Glucuronic acid, another important acid in Kombucha, facilitates the detoxification process in the body. UDP-glucuronic acid is formed in the liver of all animals and conjugates toxins for subsequent elimination [3]. Andlaur *et al*. reported that potential phytochemical toxins were detoxified in mammalian tissue by conjugation with glucuronic acid [38].

## Conclusion

The present study indicates that Kombucha may repair damage caused by environmental pollutants such as TCE and may be beneficial to patient suffering from renal impairment.

## Abbreviations

DCVC: S-(1, 2-dichlorovinyl) L. cysteine; DCVC sulfoxide: S-[1-chloro-2-(glutathionyl) vinyl]-L-cysteine sulfoxide); GGT: gamma glutamyl transpeptidase; GSH: glutathione; GSH transferase: glutathione transferase; KT: Kombucha; LDH: lactate dehydrogenase; MDA: malondialdehyde, lipid peroxidation marker; NO: nitric oxide; TCE: trichloroethylene; U/L: unit per liter.

## Competing interests

Kombucha used in the present study was supplied by the microbiology lab of the National Center for Radiation Research and Technology (NCRRT), Atomic Energy Authority (Cairo, Egypt) where the author is employed.

## Authors' contributions

OAG conceived of the study design, carried out the experiments, performed statistical analysis and drafted the manuscript. The author read and approved the final version of the manuscript.
